# Artemisinin*-*Resistant *Plasmodium falciparum* with High Survival Rates, Uganda, 2014–2016

**DOI:** 10.3201/eid2404.170141

**Published:** 2018-04

**Authors:** Mie Ikeda, Megumi Kaneko, Shin-Ichiro Tachibana, Betty Balikagala, Miki Sakurai-Yatsushiro, Shouki Yatsushiro, Nobuyuki Takahashi, Masato Yamauchi, Makoto Sekihara, Muneaki Hashimoto, Osbert T. Katuro, Alex Olia, Paul S. Obwoya, Mary A. Auma, Denis A. Anywar, Emmanuel I. Odongo-Aginya, Joseph Okello-Onen, Makoto Hirai, Jun Ohashi, Nirianne M.Q. Palacpac, Masatoshi Kataoka, Takafumi Tsuboi, Eisaku Kimura, Toshihiro Horii, Toshihiro Mita

**Affiliations:** Juntendo University School of Medicine, Tokyo, Japan (M. Ikeda, M. Kaneko, S.-I. Tachibana, M. Yamauchi, M. Sekihara, M. Hashimoto, M. Hirai, T. Mita);; Ehime University, Matsuyama, Japan (B. Balikagala, T. Tsuboi);; Tokyo Women’s Medical University, Tokyo (M. Sakurai-Yatsushiro, N. Takahashi);; National Institute of Advanced Industrial Science and Technology, Kagawa, Japan (S. Yatsushiro, M. Kataoka);; Med Biotech Laboratories, Kampala, Uganda (O.T. Katuro);; Gulu University, Gulu, Uganda (A. Olia, D.A. Anywar, E.I. Odongo-Aginya, J. Okello-Onen);; St. Mary’s Hospital Lacor, Gulu (P.S. Obwoya, M.A. Auma);; The University of Tokyo, Tokyo (J. Ohashi);; Osaka University, Osaka, Japan (N.M.Q. Palacpac, E. Kimura, T. Horii)

**Keywords:** Artemisinin, drug resistance, Plasmodium falciparum, Uganda, parasites, malaria, antimicrobial resistance

## Abstract

Because ≈90% of malaria cases occur in Africa, emergence of artemisinin-resistant *Plasmodium falciparum* in Africa poses a serious public health threat. To assess emergence of artemisinin-resistant parasites in Uganda during 2014–2016, we used the recently developed ex vivo ring-stage survival assay, which estimates ring-stage–specific *P. falciparum* susceptibility to artemisinin. We conducted 4 cross-sectional surveys to assess artemisinin sensitivity in Gulu, Uganda. Among 194 isolates, survival rates (ratio of viable drug-exposed parasites to drug-nonexposed controls) were high (>10%) for 4 isolates. Similar rates have been closely associated with delayed parasite clearance after drug treatment and are considered to be a proxy for the artemisinin-resistant phenotype. Of these, the *PfKelch13* mutation was observed in only 1 isolate, A675V. Population genetics analysis suggested that these possibly artemisinin-resistant isolates originated in Africa. Large-scale surveillance of possibly artemisinin-resistant parasites in Africa would provide useful information about treatment outcomes and help regional malaria control.

Despite reports of decreasing incidence and deaths from malaria, this disease remains a global health problem; in 2016, an estimated 216 million new cases and 445,000 deaths occurred (*1*). One of the main causes for the reduction of the malaria burden is the global deployment of artemisinin-based combination therapies as a first-line treatment (*1*). However, since first reported in 2007–2008 (*2*), artemisinin-resistant *Plasmodium falciparum* parasites have spread into the Greater Mekong Subregion of Southeast Asia (*3*,*4*). In western Cambodia, recent increases in the prevalence of piperaquine-resistant parasites have further reduced effectiveness of dihydroartemisinin/piperaquine (*5*).

Until now, there was no clear evidence for the emergence of *P. falciparum* artemisinin-resistant isolates in Africa (*6*). The standard for monitoring the emergence of artemisinin resistance has been the effectiveness of artemisinin-based combination therapies. In high-transmission regions, as in many parts of Africa where most persons have some immunity to malaria, partially immune persons may, however, experience some response to drug treatment even if they are infected by drug-resistant parasites (*7*,*8*). Interpretations of drug effectiveness could also be made difficult with the widely used ex vivo conventional drug-susceptibility assay (*3*,*9*,*10*) because reduced susceptibility to artemisinin is limited to the very narrow early ring stage of the parasites (*10*–*12*).

The recently developed ring-stage survival assay (RSA) can evaluate ring-stage–specific reduction of artemisinin susceptibility (*12*). In this assay, ring-stage parasites are exposed to 700 nmol/L of dihydroartemisinin for 6 h and then cultured for 66 h in the absence of the drug. Levels of artemisinin susceptibility are estimated according to the survival rate (i.e., the ratio of viable parasites exposed to 700 nmol/L dihydroartemisinin to parasites cultured simultaneously in drug-free medium). In a study of isolates from 31 persons in Cambodia, all isolates classified as artemisinin resistant by ex vivo conventional assay (having parasite clearance half-lives >5 h after artemisinin treatment [*4*,*13*]), also had an ex vivo survival rate >10% by RSA (*12*).

In Africa, few attempts have been made to detect artemisinin-resistant parasites by using ex vivo RSA. So far, only 2 studies have been reported, both of which showed the absence of *P. falciparum* isolates with survival rates >10% (*14*,*15*). Close monitoring is nevertheless valuable because parasites resistant to all widely used antimalarial drugs (chloroquine [*16*], pyrimethamine [*17*], and sulfadoxine [*18*]) have migrated from Southeast Asia to sub-Saharan Africa. Also reported in Africa is indigenous emergence of parasites, albeit all of minor lineages, resistant to these antimalarial drugs (*19*,*20*). 

During 2014–2016, to assess artemisinin sensitivity in Gulu, northern Uganda, we performed 4 cross-sectional surveys. We evaluated the emergence of artemisinin resistance by using 3 methods: ex vivo RSA for dihydroartemisinin; conventional ex vivo drug susceptibility assay; and genotyping of *PfKelch13*, the gene responsible for artemisinin resistance in Southeast Asia (*21*–*24*).

## Materials and Methods

### Study Site, Design, and Patients

**Figure 1:**
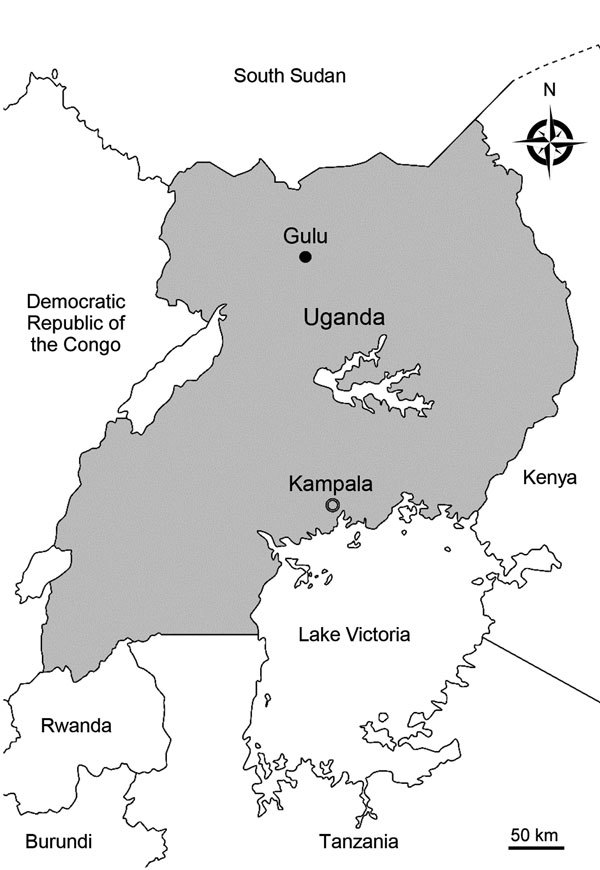
Site of study of ex vivo ring-stage *Plasmodium falciparum* survival rates, Gulu (black circle), northern Uganda, 2014–2016.

During 2014–2016, we conducted 4 cross-sectional surveys in Gulu, northern Uganda (Figure 1). In this region, malaria transmission is high; estimated prevalence is ≈60%. Each year, weak seasonal fluctuation is observed; peaks occur in May and October. All 4 surveys were performed during peak malaria seasons: October–November 2014, May–June 2015, October–November 2015, and June–July 2016. The major mosquito vector in this region is *Anopheles funestus* (*25*), and the entomological inoculation rate was estimated at >100 infective mosquito bites/person/year (*26*). Since 2004, artemether/lumefantrine has been used as a first-line treatment for uncomplicated malaria (*27*). Persons who visited St. Mary’s Hospital Lacor, Gulu, Uganda, with signs and symptoms suggestive of malaria were screened by microscopic examination of Giemsa-stained blood smears. We enrolled those with *P. falciparum* monoinfection (parasitemia >0.1%) if written informed consent was obtained from a parent/guardian (for patients <7 years of age), from a parent/guardian and from the patient (for patients 7–17 years of age), or from adult patients (>18 years of age). Ethics approval for the study was obtained from the Lacor Hospital Institutional Research and Ethics Committee (LH 021/09/13), the Ugandan National Council for Science and Technology (HS 1395), and the institutional review board at Juntendo University in Tokyo, Japan (2014168).

### Ex Vivo RSA 

We performed the ex vivo RSA as described previously, with some modifications (*12*,*28*). In brief, 1 mL of venous blood was immediately transferred to the hospital laboratory. After plasma removal, erythrocyte pellets were washed 3 times in RPMI-1640 (Thermo Fisher Scientific, Waltham, MA, USA) with 100 μg/mL gentamicin. Washed infected erythrocytes were suspended in RPMI-1640, 25 mM HEPES (4-[2-hydroxyethyl]-1-piperazineethanesulfonic acid), 2 mM l-glutamine supplemented with 100 μg/mL gentamicin and heat-inactivated 10% serum type AB from Japanese volunteers, and stored at 4°C before initiation of the ex vivo RSA. All procedures were performed within 4 h after blood collection. 

The next morning, 100 μL/well of parasite culture mixture adjusted to 2% hematocrit was dispensed with 700 nmol/L dihydroartemisinin (Tokyo Chemical Industry Co. Ltd, Tokyo, Japan). As nonexposed control, 0.1% DMSO (dimethyl sulfoxide) was used. Parasitemia >1% was adjusted to 1% by adding uninfected O-type erythrocytes. Cultures were incubated in a gas atmosphere with 5% CO_2_ and 5% O_2_ by using the AnaeroPack malaria culture system (Mitsubishi Gas Chemical Co. Inc., Tokyo, Japan). After 6 h of exposure to dihydroartemisinin, pellets were washed 3 times in RPMI-1640 with gentamicin, resuspended in fresh culture medium, and incubated for another 66 h. Assays were performed in duplicate for all samples except those obtained October–November 2014 because of insufficient sample volume. 

At the end of the culture period, by counting 20,000 erythrocytes and using thin blood smears, 2 investigators independently determined the number of viable parasites with normal morphologic appearance that developed into ring stages, trophozoites, and schizonts. Parasites that appeared pyknotic were considered to be dead (*28*,*29*). When the proportion of viable parasites in the nonexposed culture at 72 h was higher than the initial parasitemia at 0 h, the samples were considered to be interpretable (*29*). The investigators calculated survival rates as the ratios of parasites in exposed and nonexposed cultures (*12*); if the survival rates calculated by 2 researchers differed by >50%, a third researcher assessed the slides. Based on previous comparative studies of ex vivo RSA and in vivo drug susceptibility tests (*12*), the cutoff for a high survival rate by RSA was >10%. This cutoff has 89% sensitivity and 91% specificity in predicting parasites likely to have >5 h of clearance half-life after artemisinin treatment. As a reference for ex vivo RSA, we used laboratory-adapted artemisinin-susceptible (3D7) and artemisinin-resistant clones (MRA-1236 and MRA-1240) (*12*). The *P*. *falciparum* artemisinin-resistant strains IPC 3445 Pailin Cambodia 2010 (MRA-1236) and IPC 5202 Battanbang Cambodia 2011 (MRA-1240) were contributed by Didier Ménard and provided by the Malaria Research and Reference Reagent Resource Center (part of BEI Resources, National Institute of Allergy and Infectious Diseases, National Institutes of Health, Rockville, MD, USA).

### Ex Vivo Conventional Drug-Susceptibility Assay

For each sample, 100 μL of parasite culture with parasitemia adjusted to 0.05% at 2.5% hematocrit was pipetted per well into predosed culture plates and incubated under the same conditions as the ex vivo RSA for 72 h. Wells A–H were dosed with 0 (control), 0.25, 0.5, 1, 2, 4, 8, or 16 nmol/L of dihydroartemisinin, respectively. Samples were then frozen (−20°C overnight) and thawed until complete hemolysis was obtained. We assessed parasite growth by using an ELISA that quantifies parasite histidine-rich protein 2, as reported previously (*30*). We established the effective concentration of dihydroartemisinin needed to inhibit growth of *P. falciparum* by 50% (IC_50_) by nonlinear regression in the online ICEstimator software (http://www.antimalarial-icestimator.net) (*31*).

### Genotyping

We extracted parasite DNA from blood-spotted filter paper (ET31CHR; Whatman Limited, Kent, UK) by using QIAcube (QIAGEN, Hilden, Germany). The sequence of 6 propeller domains in *PfKelch13* was determined as previously reported (*32*). From isolates showing high RSA rates of survival, we determined the following 6 mutations suggested as background genetic changes for artemisinin resistance as described (*32**,**33*): D193Y in ferredoxin (*fd*), T484I in multidrug resistance protein 2 (*mdr2*), V127M in apicoplast ribosomal protein S10 (*arps10*), I356T in chloroquine resistance transporter (*crt*), V1157L in protein phosphatase (*pph*), and C1484F in phosphoinositide-binding protein (*pibp*). Samples with minor peaks >50% the height of the major peak were considered mixed genotypes.

### Whole-Genome Sequencing and Variant Detection

For 3 isolates with high survival rates by RSA, we extracted genomic DNA from leukocyte-removed blood with Acrodisc filters (Pall Corporation, New York, NY, USA) by using a QIAamp DNA Blood Mini Kit (QIAGEN). We prepared DNA libraries by using a TruSeq DNA PCR-Free Library Preparation Kit (Illumina, San Diego, CA, USA) after amplification of the genomic DNA with an Illustra GenomiPhi DNA Amplification Kit (GE Healthcare, Chicago, IL, USA) or without genome amplification with a Nextera XT DNA Sample Prep Kit (Illumina). We obtained ≈1–1.5 Gb of data per sample, consisting of paired-end reads 100 or 150 bp long by using Illumina instruments (Miseq and Hiseq2000). Data are available at the DDBJ Sequence Read Archive (http://trace.ddbj.nig.ac.jp/dra/index.html) under accession nos. DRA005346, DRA005347, and DRA005348.

For comparative analyses, we obtained raw sequence data (fastq files) of 31 *P. falciparum* isolates from various regions in Asia (n = 19) and Africa (n = 12) from the Short Read Archive at the National Center for Biotechnology Information (https://www.ncbi.nlm.nih.gov/sra) (Technical Appendix Table 1). Among 19 isolates from Asia, 6 harbored mutant alleles in *PfKelch13*. We mapped all short-read data onto the 3D7 reference genome (National Center for Biotechnology Information BioProject ID PRJNA148) by using CLC Genomics Workbench software (QIAGEN) with default parameters. We then used the resulting files for detecting single-nucleotide polymorphisms (SNPs) by using the Basic Variant Detection program in CLC Genomics Workbench. SNPs were called at all genomic positions with >80% frequency of >10 reads support.

### Population Genetic Analyses

To clarify lineages of isolates exhibiting high rates of survival according to RSA, we performed principal component analysis and STRUCTURE analysis (*34*). SNPs with minor allele frequency >25% were used after standardization. For principal component analysis, we used BellCurve for Excel (Social Survey Research Information Co., Ltd, Tokyo, Japan). We used STRUCTURE 2.3.3 to assign individual isolates from all populations to 2 clusters (i.e., K = 2). For each run, a burn-in period of 10,000 steps was followed by 10,000 iterations under the admixture model and the assumption of uncorrelated haplotype frequencies among populations.

## Results

### Ex Vivo RSA

In the ex vivo RSA study, we enrolled 249 patients (Table 1). Most patients were children; median age was 3 years. Fever >37.5°C was reported for 83% of patients. Other signs and symptoms were present for 7%–22% of enrolled patients. Examination of blood smears and species-specific PCR revealed that all blood samples contained monoinfections with *P. falciparum*.

**Table 1:** Baseline characteristics of patients enrolled in study of *Plasmodium falciparum* ex vivo RSA survival rates, Uganda, 2014–2016*

Characteristics	Enrolled	RSA results obtained
No. patients	249	194
2014 Oct	16	15
2015 May	74	43
2015 Oct	63	51
2016 June	96	85
Sex, no. patients		
M	126	92
F	123	102
Age, y		
Median (IQR)	3.0 (2.0–4.5)	2.9 (2.0–4.4)
Fever >37.5°C		
No. (%) patients	207 (83)	177 (92)
Median (IQR) body temperature, °C	39 (38.0–39.4)	39 (38.3–39.5)
Signs and symptoms, % patients		
Cough	22	29
Vomiting	21	25
Abdominal pain	11	21
Headache	10	19
Diarrhea	7	10
Convulsion	7	8
Day 0 parasitemia, median (IQR), %	1.71 (0.17–4.13)	1.95 (0.85–4)

*IQR, interquartile range; RSA, ring-stage survival assay.

We obtained successful ex vivo RSA results for dihydroartemisinin for 194 (77.9%) patients. Unsuccessful results were mostly caused by insufficient parasite growth (n = 28) or inability to count 20,000 erythrocytes because of low-quality blood smears and limited amounts of blood (n = 27). We found no significant differences for patient background characteristics or parasitemia in all enrolled and ex vivo RSA–successful patients (Table 1; Technical Appendix Table 2). Artemisinin-susceptible laboratory clone 3D7 showed no parasites at 700 nmol/L, whereas artemisinin-resistant laboratory clones MRA-1236 and MRA-1240 showed survival rates (± SEM) of >10% (14.3 ± 0.5% and 27.0 ± 2.7%, respectively) (Table 2).

**Table 2:** Characteristics of *Plasmodium falciparum* isolates with high ex vivo ring-stage survival rates and patients enrolled in study of *Plasmodium falciparum* survival rates, Uganda, 2014–2016*

Sample name	Patient age, y	Patient sex	Signs and symptoms	Day 0 parasitemia, %	Early ring-stage parasites at day 0, %	Mean parasite survival rate, %, ± SEM
H1	3.0	M	Fever, headache, cough	0.85	80.4	34.3
H2	2.0	M	Fever	15.0	98.2	13.3 ± 1.5
H3	3.2	M	Fever	0.76	51.9	18.9 ± 0.9
H4	4.5	F	Fever	2.56	72.0	18.1 ± 1.4
3D7	NA	NA	NA	NA	NA	0 ± 0
MRA-1236	NA	NA	NA	NA	NA	14.3 ± 0.5
MRA-1240	NA	NA	NA	NA	NA	27.0 ± 2.7

*Survival rate, parasitemia at 700 nmol/L dihydroartemisinin exposed/parasitemia at 0 nmol/L control × 100; mean value ± SEM in 2 (H2, H3, and H4) and 3 (3D7, MRA-1236, and MRA-1240) independent trials. Day 0, day of enrollment. NA, not applicable.

**Figure 2:**
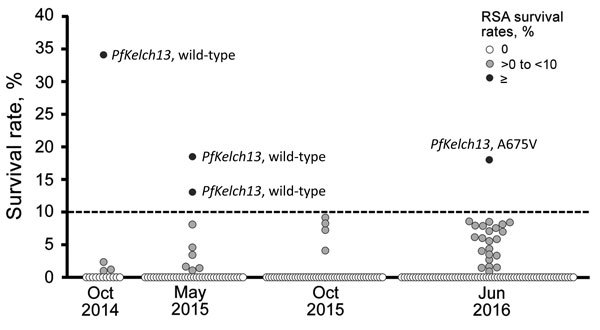
Distribution of ex vivo ring-stage *Plasmodium falciparum* survival rates, by RSA during each study period, 2014–2016. Susceptibility to dihydroartemisinin was determined by using ex vivo RSA survival rates. Survival rate was calculated as follows: (parasitemia at 700 nmol/L dihydroartemisinin exposed/parasitemia at 0 nmol/L control) × 100. Broken line indicates the cutoff value for what we consider to be high ex vivo RSA survival rates (>10%). RSA, ring-stage survival assay.

Among the study samples, 4 (2.1%) were classified as having high parasite survival rates by RSA; mean survival rates (± SEM) ranged from 13.3% ± 1.5% (isolate H2) to 34.3% (isolate H1) (Figure 2, Table 2). We assayed all except 1 isolate (H1) in duplicate. We observed these resistant isolates in all sampling periods except October–November 2015. One isolate (H1) had higher survival rates (34.3%) than the 2 artemisinin-resistant laboratory clones from Cambodia (MRA-1236 and MRA-1240). Developmental stages of the parasites at enrollment were microscopically determined (Technical Appendix Figure). In the 3 isolates with high survival rates by RSA, proportions of early ring-stage parasites at enrollment were high (72%–98%) (Technical Appendix Table 3), except for 1 isolate (H3), which had a lower proportion of early ring-stage parasites. For parasites with survival rates ≈10% (I1, I5, and I7), early ring-stage parasites were ≈51.9%–57.7% and the rest were late ring-stage parasites. These observations show that the developmental stages of the parasite before start of the RSA was not biased or skewed so as to influence RSA results. In fact, survival rates of samples with an almost 50:50 ratio of parasites in early and late stages might be underestimated because susceptibility to artemisinin occurs only during the early ring stage (*10*–*12*).

### Conventional Ex Vivo Drug Susceptibility Assay for Dihydroartemisinin

**Figure 3:**
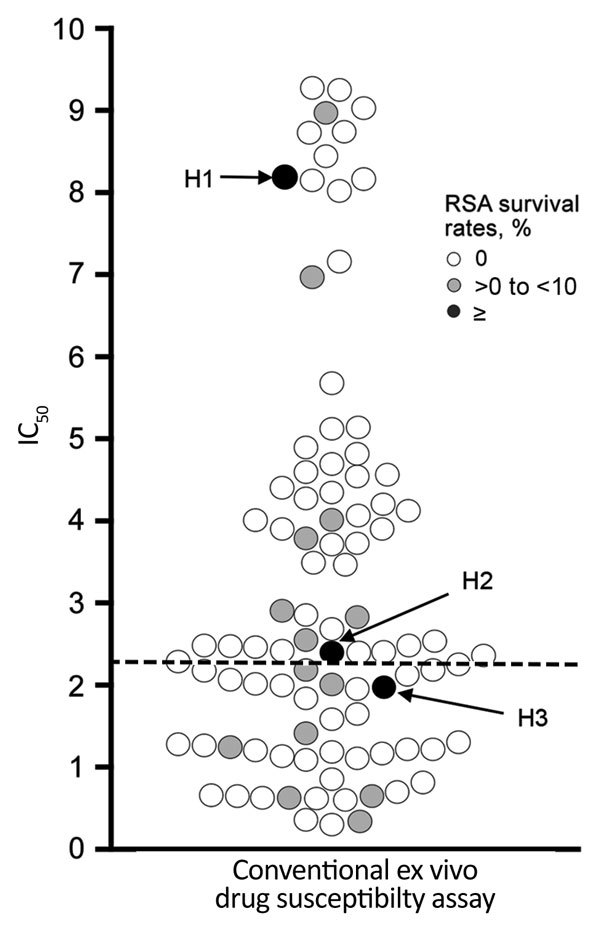
Distribution of IC_50_ of *Plasmodium falciparum* values by dihydroartemisinin in conventional ex vivo drug susceptibility assay. IC_50_ values to dihydroartemisinin in conventional ex vivo drug susceptibility assay are plotted. Geometric mean IC_50_ values in isolates with survival rates of 0, >0 to <10%, and >10% (high RSA survival) were 3.0 nmol/L (n = 74), 1.9 nmol/L (n = 14), and 3.3 nmol/L (n = 3), respectively. In the conventional ex vivo drug susceptibility assay, replication of parasites was monitored after continuous exposure to dihydroartemisinin for 72 h. Dashed line indicates geometric mean IC_50_ value of all isolates (2.25 nmol/L). IC_50_, concentration needed to inhibit 50% growth; RSA, ring-stage survival assay.

We performed a conventional ex vivo dihydroartemisinin-susceptibility assay in all but 1 survey (June–July 2016) and successfully determined ex vivo RSA and conventional ex vivo IC_50_ values for 93 patients (Figure 3). IC_50_ values were not correlated with survival rates by RSA. Geometric mean IC_50_ for isolates with high survival rates by RSA (3.3 nmol/L) were similar to those for the artemisinin-susceptible isolates (2.25 nmol/L).

### Genotypes of *PfKelch13* and Potential Background Mutations

Only 5 (2.6%) isolates (C469Y, M472V, A621S, V666I, A675V) harbored nonsynonymous mutations at propeller domains in *PfKelch13*, all of which were observed as singletons (Technical Appendix Table 4). Among 4 parasites with high survival rates by RSA, 1 harbored the A675V mutation and the other 3 harbored the wild-type allele (Figure 2). We observed no background genetic changes for artemisinin resistance (D193Y in *fd*, T484I in *mdr2*, V127M in *arps10*, I356T in *crt*, V1157L in *pph*, and C1484F in *pibp*; data not shown).

### Lineages of Parasites with High Survival Rates by RSA

**Figure 4:**
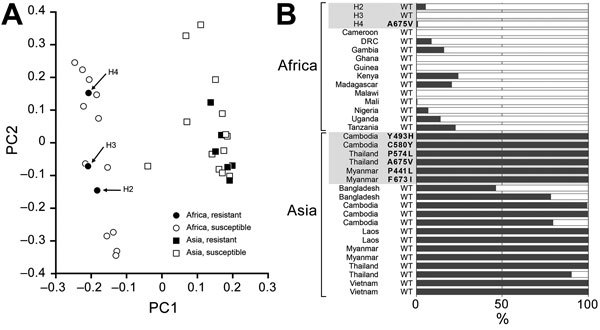
Potential lineage of ring-stage *Plasmodium falciparum* isolates with high survival rates according to ring-stage assay (RSA) in Uganda. Principal component analysis (A) and STRUCTURE (*34*) analysis (B) suggested an indigenous emergence of parasites with high survival rates by RSA (H2, H3, and H4) in Africa. *P. falciparum* isolates (n = 31) that originated from Asia (n = 19), including 6 isolates with *PfKelch13* mutation, or from Africa (n = 12) were obtained from Sequence Read Archive in National Center for Biotechnology Information (https://www.ncbi.nlm.nih.gov/sra). DRC, Democratic Republic of the Congo; PC1, first principal component; PC2, second principal component; WT, wild-type.

To clarify whether parasites with high survival rates by RSA indigenously originated in Africa or migrated from Southeast Asia, we performed principal component and STRUCTURE analyses. Whole-genome sequences were determined for all except 1 (H1) parasite isolate with a high survival rate by RSA. As reference, we used 31 *P. falciparum* isolates with origins from Asia or Africa. High-quality whole-genome sequence data enabled identification of 174,266 SNPs, of which 168,908 were biallelic SNPs. Among these, we used 14,341 SNPs with minor allele frequency >25% for the analyses. In principal component analysis, the first principal component clearly separated isolates from Africa from those from Southeast Asia (Figure 4, panel A). All 7 reference isolates harboring mutant *PfKelch13* were located in the cluster from Southeast Asia. In contrast, all 3 isolates from Uganda with high survival rates by RSA were inside the cluster from Africa. STRUCTURE analysis also clarified the distinct population structure of isolates from Southeast Asia compared with all isolates from Africa, including the 3 isolates with high survival rates by RSA (Figure 4, panel B).

## Discussion

Emergence and spread of artemisinin-resistant *P. falciparum* outside the Greater Mekong Subregion pose a serious public health threat, especially in sub-Saharan Africa, the region that in 2015 accounted for 90% of global malaria cases and 92% of malaria deaths (*35*). The recently developed ex vivo RSA found that 4 (2.1%) isolates from Uganda showed high (>10%) survival rates, levels of which are reported to be closely associated with slow-clearing *P. falciparum* infections (*12*). Among these, mutation in *PfKelch13* was observed in only 1 isolate. Population genetic analysis with whole-genome sequences demonstrated that these isolates belong to the cluster from Africa, suggesting an indigenous origin rather than migration from Southeast Asia.

So far, 2 ex vivo RSA results have been reported in sub-Saharan Africa: in Cameroon (*15*) and in Kampala, Uganda, ≈300 km from our study site (*14*). Neither study found isolates with high survival rates. In our analysis, 4 (2.1%) isolates had survival rates >10%. In addition, 3 (1.5%) isolates with survival rates of ≈10% had lower proportions of early ring-stage parasites (51.9%–57.7%) at patient enrollment. Because artemisinin resistance is known to be confined to the early ring stage (*10*–*12*), the observed survival rate for these isolates might be underestimated. For our analysis, we assumed that an overall 2%–4% of isolates had artemisinin-resistant potential. Since the discovery of *PfKelch13* as an artemisinin-resistant responsible gene in 2013 (*21*), numerous molecular epidemiologic studies identified >200 mutations in this gene (*4*,*35**,**36*). The correlation with clinical and laboratory artemisinin resistance in 6 mutations (N458Y, Y493H, R539T, I543T, R561H, C580Y) has been validated (*37*). In our study, 1 isolate with a high survival rate by RSA harbored a mutation allele in *PfKelch13* (A675V). This mutation is reported to have a level of parasite clearance half-life similar to that of the most prevalent C580Y mutation (*4*,*38*) and has thus been regarded as candidate artemisinin-related mutation (*37*). The allele is distributed in Southeast Asia (Thailand–Myanmar border) and sub-Saharan Africa (Democratic Republic of the Congo and Rwanda) (*4*,*38*–*40*). However, our population genomic analysis revealed that this isolate belongs to African lineage, suggesting that artemisinin-resistant migration of A675V from Southeast Asia to Africa is less likely than the indigenous emergence of this mutation in Africa.

Three other isolates with high survival rates by RSA harbored a wild-type allele in *PfKelch13*. A similar finding in Cambodia has been recently reported (*41*). The parasiticidal mechanism of artemisinin is known to result from induction of highly reactive carbon-centered radicals that inactivate multiple crucial proteins (*42*). To combat this effect, resistant parasites are believed to enhance the oxidative stress response, altering the cell cycle (*29**,**36**,**39**,**43**–**45*). Recent population transcriptome analyses that used 1,043 clinical *P. falciparum* isolates obtained directly from patients with acute falciparum malaria demonstrated associations between *PfKelch13* mutations and upregulation of unfolded protein response, one of the stress response systems, and PI3K/PI3P/AKT pathways (*46*). One possible hypothesis to explain the absence of a *PfKelch13* mutation in the ex vivo RSA artemisinin-resistant isolates is the activation of alternate pathways by yet unknown mechanism(s).

Witkowski et al. showed a strong correlation between ex vivo RSA survival rates and in vivo parasite clearance half-lives in Cambodia (*12*). When the ex vivo RSA survival rate cutoff of 10% is applied to their study, 29 of 30 infections are accurately identified as artemisinin resistant and artemisinin susceptible (*12*). This cutoff point produced 89% sensitivity and 91% specificity for in vivo artemisinin resistance in the parasites from Cambodia (*12*). However, a similar correlation study is lacking for isolates from Africa. Because many factors such as levels of host immunity and pharmacokinetics could complicate drug clinical effectiveness, further correlation of ex vivo RSA and in vivo studies is strongly required in the various malaria-endemic regions with different population ethnicities and malaria ecologies.

Our study has several limitations. Parasites were not cryopreserved; thus, survival rate was not reconfirmed by in vitro RSA with culture-adapted parasites. Reconfirmation was not performed because of the difficulties in logistics and cryopreservation of natural parasites at our field site and transportation to our laboratory. However, we performed ex vivo RSA duplicate (except during October–November 2014), enabling confirmation via independently performed assays. In addition, we did not obtain clinical confirmation of artemisinin resistance for all 4 isolates with high survival rates by RSA because clinical study in 2014 was performed only during October–November, and only in 9 random isolates was artemether/lumefantrine treatment efficacy assessed.

In the conventional ex vivo artemisinin susceptibility assay, parasites are exposed to dihydroartemisinin for 48–72 h, affecting trophozoite and schizont stages. Strictly, therefore, this assay theoretically underestimates the level of artemisinin resistance. Using ex vivo assays, we found that the mean IC_50_ in isolates with high survival rates by RSA was similar to that for those isolates characterized as having low survival rates or those that are completely susceptible (showed no survival by RSA). This finding was similar to previous field observations in Cambodia, where a lack of correlation was found between ex vivo survival rates by RSA and IC_50_s to dihydroartemisinin in conventional ex vivo drug susceptibility assay (*12*), supporting the notion that conventional ex vivo assays may not be useful for properly distinguishing isolates with decreased artemisinin susceptibility in the field.

In conclusion, our analysis has revealed the potential emergence of *P*. *falciparum* with high survival rates by RSA in Uganda. One of these parasites harbored a *PfKelch13* A675V mutation. Because ≈90% of malaria cases occur in Africa (*35*), emergence and spread of artemisinin resistance impose substantial obstacles for effective malaria control and the approach toward elimination. It is thus imperative to perform further intensive surveillance for artemisinin resistance in various malaria-endemic regions in Africa and to elucidate genetic changes that confer resistance to artemisinin in parasites in Africa. Use of ex vivo RSA would help solve these challenges.

Technical Appendix Additional information about detection of artemisinin-resistant *Plasmodium falciparum* isolates by using ex-vivo ring-stage survival assay in Uganda.
