# Supplementary material for: Artemisinin-Resistant Plasmodium falciparum with High Survival Rates, Uganda, 2014–2016
Source: Emerg Infect Dis. 2018 Apr;24(4):718–26. doi: 10.3201/eid2404.170141 (PMC5875287; doi:10.3201/eid2404.170141)
Supplement: Technical Appendix — Additional information about detection of artemisinin-resistant Plasmodium falciparum isolates by using ex-vivo ring-stage survival assay in Uganda. [file 17-0141-Techapp-s1.pdf]

Article DOI: https://doi.org/10.3201/eid2404.170141

# Artemisinin-Resistant *Plasmodium falciparum* with High Survival Rates, Uganda, 2014–2016

## Technical Appendix

## Detection of artemisinin-resistant *Plasmodium falciparum* isolates using ex-vivo ring-stage survival assay in Uganda

**Technical Appendix Table 1.** *Plasmodium falciparum* isolates used for PCA and STRUCTURE analysis

| ID* | Mutation in *PfKelch13* | Country | Region | Read Archive ID† |
|---|---|---|---|---|
| H2 | WT | Uganda | Africa | DRA005347 |
| H3 | WT | Uganda | Africa | DRA005346 |
| H4 | A675V | Uganda | Africa | DRA005348 |
| 1 | WT | Cameroon | Africa | ERS418899 |
| 2 | WT | D.R.Congo | Africa | ERS347631 |
| 3 | WT | Gambia | Africa | ERS157495 |
| 4 | WT | Ghana | Africa | ERS010122 |
| 5 | WT | Guinea | Africa | ERS041976 |
| 6 | WT | Kenya | Africa | ERS087025 |
| 7 | WT | Madagascar | Africa | ERS404077 |
| 8 | WT | Malawi | Africa | ERS032647 |
| 9 | WT | Mali | Africa | ERS010184 |
| 10 | WT | Nigeria | Africa | ERS347697 |
| 11 | WT | Uganda | Africa | ERS045919 |
| 12 | WT | Tanzania | Africa | ERS013057 |
| 13 | Y493H | Cambodia | Asia | ERS028717 |
| 14 | C580Y | Cambodia | Asia | ERS023736 |
| 15 | P574L | Thailand | Asia | ERS142852 |
| 16 | A675V | Thailand | Asia | ERS142845 |
| 17 | P441L | Myanmar | Asia | ERS143489 |
| 18 | F673I | Myanmar | Asia | ERS086879 |
| 19 | WT | Bangladesh | Asia | ERS010785 |
| 20 | WT | Bangladesh | Asia | ERS174561 |
| 21 | WT | Cambodia | Asia | ERS032109 |
| 22 | WT | Cambodia | Asia | ERS050881 |
| 23 | WT | Cambodia | Asia | ERS338591 |
| 24 | WT | Laos | Asia | ERS086919 |
| 25 | WT | Laos | Asia | ERS174612 |
| 26 | WT | Myanmar | Asia | ERS347690 |
| 27 | WT | Myanmar | Asia | ERS086951 |
| 28 | WT | Thailand | Asia | ERS142855 |
| 29 | WT | Thailand | Asia | ERS347665 |
| 30 | WT | Vietnam | Asia | ERS087028 |
| 31 | WT | Vietnam | Asia | ERS174549 |

*H2, H3 and H4 are categorized as high-RSA survival (survival rate ≥10%).
†Read data are registered on Sequence Read Archive at National Center for Biotechnology Information (https://www.ncbi.nlm.nih.gov/sra)

**Technical Appendix Table 2.** Background information of *Plasmodium falciparum* isolates in Uganda according to RSA results*

| | Parasite phenotype grouping based on ex-vivo RSA (survival rate) | | |
|---|---|---|---|
| Characteristics | High-RSA survival (≥10%) | Intermediate-RSA survival (>0% but <10%) | No-RSA survival (0%) |
| Patients (n) | | | |
| 2014 Oct | 1 | 3 | 11 |
| 2015 May | 2 | 6 | 35 |
| 2015 Oct | 0 | 6 | 45 |
| 2016 June | 1 | 22 | 62 |
| Gender (n) | | | |
| Male | 3 | 18 | 71 |
| Female | 1 | 19 | 82 |
| Age (year) | | | |
| Median (IQR*) | 3.1 (2.0–4.5) | 2.4 (1.7–4.0) | 3.0 (2.0–4.4) |
| Fever (≥37.5°C) | | | |
| Number (%) | 4 (100) | 35 (95) | 140 (92) |
| Median (IQR*) | 38.9 (38.7–39.0) | 39.5 (38.7–39.6) | 39.0 (38–39.5) |
| Symptoms (n) | | | |
| Cough | 33 | 29 | 29 |
| Headache | 33 | 19 | 19 |
| Vomiting | 0 | 25 | 25 |
| Abdominal pain | 0 | 21 | 21 |
| Diarrhea | 0 | 10 | 10 |
| Convulsion | 0 | 8 | 8 |
| Day 0 parasitemia | | | |
| Median (IQR) | 1.79% (0.83%–5.63%) | 1.92% (1.11%–3.20%) | 1.96% (0.80%–4.0%) |

*IQR: Interquartile range

**Technical Appendix Table 3.** Proportion of early ring-stage parasites at enrollment

| Sample ID | Proportion of early ring-stage parasite (%)* | Survival rate (%)† | Sampling period |
|---|---|---|---|
| H1 | 80.4 | 34.3 | 2014, Oct |
| H2 | 98.2 | 13.3 | 2015, May |
| H3 | 51.9 | 18.9 | 2015, May |
| H4 | 72.0 | 18.1 | 2016, June |
| I1 | 57.7 | 9.6 | 2015, Oct |
| I2 | 92.3 | 8.7 | 2016, June |
| I3 | 68.6 | 8.6 | 2016, June |
| I4 | 98.0 | 8.5 | 2016, June |
| I5 | 52.8 | 8.4 | 2015, Oct |
| I6 | 100.0 | 8.2 | 2015, May |
| I7 | 51.9 | 8.2 | 2016, June |
| I8 | 100.0 | 8.0 | 2016, June |
| I9 | 67.3 | 8.0 | 2016, June |
| I10 | 92.0 | 7.6 | 2016, June |
| I11 | 100.0 | 7.4 | 2015, Oct |
| I12 | 58.5 | 7.2 | 2016, June |
| I13 | 84.0 | 7.1 | 2016, June |
| I14 | 100.0 | 6.2 | 2016, June |
| I15 | 98.2 | 6.1 | 2016, June |
| I16 | 100.0 | 5.9 | 2016, June |
| I17 | 100.0 | 5.7 | 2016, June |
| I18 | 100.0 | 4.7 | 2015, May |
| I19 | 74.0 | 4.5 | 2016, June |
| I20 | 100.0 | 4.3 | 2015, Oct |
| I21 | 91.8 | 4.1 | 2016, June |
| I22 | 31.5 | 3.6 | 2016, June |
| I23 | 100.0 | 3.6 | 2015, May |
| I24 | 94.6 | 3.4 | 2016, June |
| I25 | 57.1 | 2.8 | 2016, June |
| I26 | 95.7 | 2.5 | 2014, Oct |
| I27 | 100.0 | 1.8 | 2015, May |
| I28 | 96.0 | 1.8 | 2016, June |
| I29 | 97.0 | 1.6 | 2016, June |
| I30 | 100.0 | 1.6 | 2015, May |
| I31 | 73.7 | 1.6 | 2016, June |
| I32 | 90.0 | 1.3 | 2015, May |
| I33 | 100.0 | 1.2 | 2015, May |

| Sample ID | Proportion of early ring-stage parasite (%)* | Survival rate (%)† | Sampling period |
|---|---|---|---|
| I34 | 100.0 | 1.1 | 2014, Oct |
| I35 | 100.0 | 1.0 | 2016, June |
| I36 | 100.0 | 0.7 | 2015, Oct |
| I37 | 24.0 | 0.2 | 2015, Oct |

*Rate of early ring-stage parasite = number of early ring form / total number of parasite × 100.
†Survival rate = parasitemia at 700 nmol/L dihydroartemisinin exposed / parasitemia at 0 nmol/L control × 100.

**Technical Appendix Table 4.** Prevalence of *PfKelch13* mutation in samples where ex-vivo RSA was successfully performed

| | Parasite phenotype based on ex-vivo RSA | | |
|---|---|---|---|
| Mutation in *PfKelch13* | High-RSA survival (≥10%) | Intermediate-RSA survival (>0% but <10%) | No-RSA survival (0%) |
| Wild type | 3 | 32 | 144 |
| C469Y | 0 | 1 | 0 |
| M472V | 0 | 0 | 1 |
| A621S | 0 | 0 | 1 |
| V666I | 0 | 0 | 1 |
| A675V | 1 | 0 | 0 |
| Unknown* | 0 | 4 | 6 |

*No sequence data were obtained.

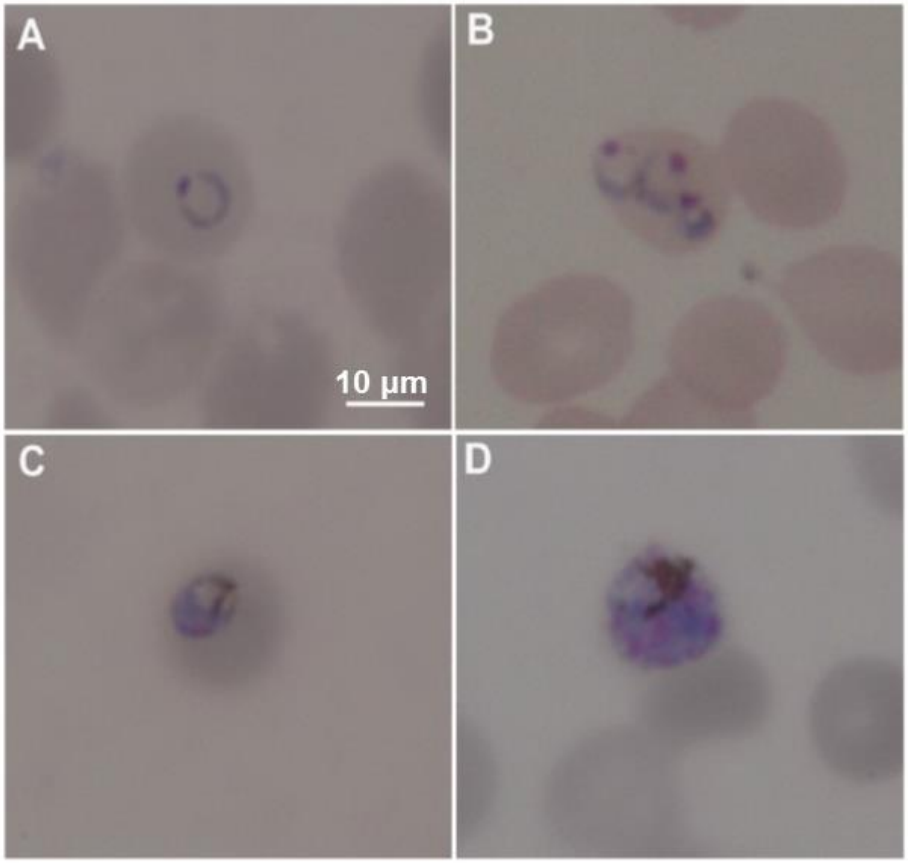


**Technical Appendix Figure**. Representative images of high-RSA survival isolates. Survived parasites developed into A) Late ring-form, B) Early trophozoite, C) Mid-trophozoite, or D) Schizont after 6 h dihydroartemisinin pulse at 700 nmol/L, followed by 66 h culture in drug-free medium. Photographs were obtained from Giemsa stained thin blood smears of H2 (A, B) and H3 (C, D) parasites.
